# Association between preoperative hemoglobin with length of hospital stay among non-cardiac and non-obstetric surgery patients: a secondary analysis of a retrospective cohort study

**DOI:** 10.1186/s13019-024-02566-5

**Published:** 2024-02-16

**Authors:** Zhaopeng Wang, Min Liu, Hengtong Men, Chunfeng Lyu, Ning Zheng

**Affiliations:** 1 Department of General Medicine, Tianjin Beichen Hospital, Beichen District, Tianjin, 300400 China; 2 Department of Clinical Laboratory, Tianjin Beichen Hospital, Beichen District, Tianjin, 300400 China

**Keywords:** Preoperative hemoglobin, Length of stay, Multivariate analysis, Retrospective studies

## Abstract

**Background:**

Previous studies concerning the association between preoperative Hemoglobin (HB) level and the Length Of hospital Stay (LOS) in patients with non-cardiac surgery and non-obstetric surgery remain inconclusive. Herein, the objective of this study was to analyze whether and to what extent the preoperative HB level was connected with the LOS in non-cardiac and non-obstetric surgery patients.

**Methods:**

This retrospective cohort study was performed at a single institution, involving patients who underwent elective non-cardiac, non-obstetric surgery from April 2007 to September 2013. Clinical characteristics of patients such as demographics, comorbidities, preoperative HB level, LOS, mortality, procedure length, and pulmonary hypertension (PHTN) Severity Class data were collected. A univariate analysis was used to determine the association between clinical characteristics and LOS. Multivariate regression analysis was conducted to investigate the relationship between preoperative HB level and LOS.

**Results and discussion:**

In this study, 311 patients were included. We observed that compared with the LOS > 7 days group, the average HB level of patients in the LOS ≤ 7 days group was higher (12.04 ± 2.20 g/dl vs. 10.92 ± 2.22 g/dl, *p* < 0.001). In addition, there were fewer patients with moderate-to-severe anemia in LOS ≤ 7 days group than the LOS > 7 days group (32.74% vs 58.82%, *p* < 0.001). In addition, we found that patients with LOS ≤ 7 days were accompanied with lower mortality (0.44% vs. 7.06%, *p* < 0.001) and lower mean combined pulmonary artery systolic pressure (PASP) and right ventricular systolic pressure (RVSP) than that in patients with LOS > 7 days (42.56 ± 11.97 vs. 46.00 ± 12.37, *p* < 0.05). After controlling for relevant confounders, we discovered a nonlinear association between preoperative HB level and LOS as well as a threshold effect based on LOS. Specifically, when preoperative HB level was less than 11.9 g/dL, LOS decreased by 2 days for each 1 g/dL increase in HB level. However, LOS did not alter substantially with the rise of preoperative HB level when it was higher than 11.9 g/dL.

**Conclusion:**

Our study showed a close non-linear association between preoperative HB level and LOS in patients with non-cardiac surgery and non-obstetric surgery. In particular, for patients with preoperative HB less than 11.9 g/dL, increasing the preoperative HB level can help shorten the LOS after operation.

**Supplementary Information:**

The online version contains supplementary material available at 10.1186/s13019-024-02566-5.

## Introduction

Anemia is a clinical common phenomenon with different incidence rate owing to the different diseases and different definition standards. A systematic review of surgical anemia showed the incidence of preoperative anemia ranging from 5 to 76% [1]. In addition, it was reported that the incidence of preoperative anemia was 24–28.1% in patients undergoing cardiac surgery [2, 3], whereas 28.7–30.44% in non-cardiac surgery patients [4, 5].

Previous studies have demonstrated the association between preoperative anemia and adverse clinical outcomes, including prolonged hospitalization [6–8], increased mortality, and more serious complications [2, 9]. Among these, LOS has attracted a lot of attention in recent years as it is closely related to the patient's economic burden and quality of life. Indeed, numbers of studies have analyzed the effects of patient population data, comorbidities, preoperative laboratory indicators, and perioperative related factors, on LOS [10–12]. However, few reports have addressed the effect of preoperative HB levels on LOS, especially in patients undergoing non-cardiac surgery and non-obstetric surgery.

Although some previous studies have analyzed the relation between preoperative HB and LOS in patients undergoing noncardiac surgery, most of them only analyzed the impact of patient population data, comorbidities, preoperative laboratory indicators, and perioperative related factors on LOS, but did not analyze the impact of PHTN on LOS. However, it should be pointed out that PHTN is common in surgical patients, and the perioperative surgical risk of such patients is higher than that of patients without PHTN, which may indicate that such patients will have a longer postoperative LOS. This study is based on a secondary analysis of previously published research data, and adds preoperative PHTN factors to previous studies to explore whether preoperative HB is still independently associated with LOS in patients with non-cardiac surgery and non-obstetric surgery. The threshold value of the impact of HB level on LOS can better illustrate the correlation between the two.

Taken these into consideration, this study conducted a secondary analysis based on previously published data, aimed to investigate whether and to what extent preoperative HB level was related to LOS in patients undergoing non-cardiac surgery and non-obstetric surgery.

## Materials and methods

### Study population

**Fig. 1:**
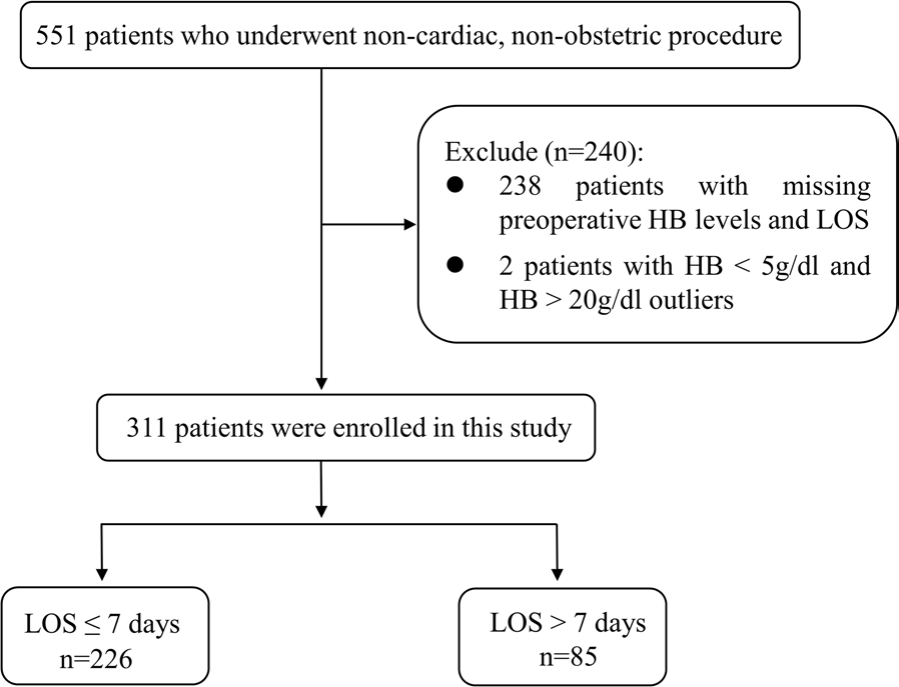
Flowchart of recruitment and research. Flowchart of the patient enrollment process in this study. A total of 311 patients were included in this study. In total, 226 patients were subgrouped into the LOS ≤ 7 days group and 85 patients were subgrouped into the LOS > 7 days group

Patients (age ≥ 18) who underwent elective non-cardiac, non-obstetric surgery under general anesthesia at a single institution (University of Washington Medical Center) from April 2007 to September 2013 were retrospectively investigated [13]. In this study, we collected data from patient charts, including those admitted on the same day and those awaiting surgery. When patients had undergone multiple surgeries during a single hospital stay, we included only data from the initial surgery. Cases were excluded if: (1) data from two-dimensional echocardiography (ECHO) within 1 year prior to surgery were missing; (2) the patient was admitted to an inpatient ward or ICU bed > 24 h prior to the procedure; (3) there was absent or incomplete pre-procedure anesthesia clinic visit data; (4) the procedure or surgery was cancelled before or after administration of anesthesia. After excluding 238 patients with preoperative HB levels and loss of LOS and 2 patients with HB data outliers, 311 patients were finally enrolled for date analysis (Fig. 1). The clinic-pathological features of the patients, such as age, gender, pre-procedure BMI, resting heart rate (HR), preoperative systolic (SBP) and diastolic blood pressure (DBP), tobacco use, preoperative comorbidities such as systemic hypertension, coronary artery disease (CAD), congestive heart failure (CHF), arrhythmia, diabetes mellitus (DM), venous thromboembolism (VTE), asthma, chronic obstructive pulmonary disease (COPD), obstructive sleep apnea (OSA), and renal failure, preoperative laboratory indicators, such as creatinine level, and room air arterial oxygen saturation (SaO_2_), were collected. The other data obtained by ECHO including left ventricular ejection fraction (EF), PASP, and RVSP, were also collected and analyzed. This project was approved by the University of Washington Institutional Review Board.

In this study, we first conducted a single-factor logistic regression analysis between each covariate and LOS to clarify the impact of each covariate on LOS, as shown in Table 2. Then we further screen for confounding factors that affect the relationship between HB and LOS (Additional file 1: Table S1–S4). The criteria for screening confounding factors are as follows: (1) The impact of introducing covariates in the basic model or removing covariates from the complete model on the regression coefficient of HB is > 10%; (2) The *p value* of the regression coefficient of the covariate on LOS is < 0.1; (3) variables meeting criteria 1 or 2 were used as confounders. Screened confounding factors include gender, American Society of Anesthesiologists (ASA) classification, procedure length, combined PASP and RVSP, mortality, intrathoracic, systemic hypertension, venous thromboembolism, asthma, renal failure (serum creatinine > 1.5 mg/dl), and heart rate. Multiple logistic regression analysis was used to further analyze the true relationship between HB and LOS without adjusting confounding variables and confounding factors. Different models in the data were adjusted based on the screened-out covariates. They are models that do not adjust any variables, Model I (only adjust the mortality variable), and Model II (only adjust all variables filtered out by criterion 1 or criterion 2). Additional file 1: Tables S1–S4 show the specific screening process. Smooth curve fitting was used to observe whether there was a nonlinear relationship between preoperative HB levels and LOS. When nonlinear associations were found, a two-piece linear regression model was applied to test for threshold effects.

### Study parameters

Based on the World Health Organization (WHO) anemia standard [14], the preoperative HB level was divided into non-anemia group (male HB > 13 g/dL, female HB > 12 g/dL), mild anemia group (female HB 11–12 g/dL, male HB 11–13 g/dL) and moderate-to-severe anemia group (HB < 11 g/dL). In addition, variables related to anesthesia and surgery were included, such as ASA class, procedure length, and mortality. Data from ECHO performed within a year of the index surgical date were collected. As for the severity of PHTN, it was divided into none PHTN (PASP or RVSP < 36 mmHg), mild PHTN (36 mmHg ≤ PASP or RVSP ≤ 50 mmHg), and moderate-to-severe PHTN (PASP or RVSP > 50 mmHg) [15, 16]. Besides, LOS was defined as the interval from the date of surgery until the date of hospital discharge or in-hospital death. Prolonged LOS was defined as more than 7 days, which corresponds to the > 75th centile LOS of the data.

### Statistical methods

Anonymized raw data were uploaded by Shah, Aalap C. et al. at the Datadryad Web site (www.datadryad.org) with ownership authorized [13]. Therefore, we further analyzed such data on a different hypothesis without violating the authors’ rights.

All statistical analyses were conducted using the statistical software package R (http://www.R-project.org, The R Foundation) and Empower-Stats (http://www.empowerstats.com, X&Y Solutions, Inc., Boston, MA). Normally distributed continuous variables are expressed as mean ± standard deviation (SD), and non-normally distributed continuous variables are replaced by median and interquartile range (IQR). Categorical variables are presented as totals and percentages. Multivariate multiple imputation with chained equations was used to impute missing data. Univariate analysis was used to determine the relation between clinical characteristics and LOS. Multivariate regression analysis was conducted to investigate the relation between preoperative HB level and LOS. A threshold, nonlinear association between HB and LOS was found in a generalized additive model (GAM). A *p* value < 0.05 was considered statistically significant.

## Results

### Clinical characteristics of included patients

A total of 311 participants (151 males and 160 females) were selected for the final data analysis. Table 1 listed the baseline characteristics, operation-related and prognostic data of the selected participants according to LOS status. There were 226 (72.67%) patients in LOS ≤ 7 days group and 85 patients in LOS > 7 days group. No statistically differences were detected in age, gender, BMI, SBP, DBP, tobacco, white blood cell count, room air SaO_2_, serum creatinine, EF value between the two groups, respectively. In addition, preoperative comorbidities such as angina, coronary artery disease, arrhythmia, asthma, obstructive sleep apnea, renal failure (serum creatinine > 1.5 mg/dL), COPD and diabetes also showed no statistical difference between the two group. However, the mean preoperative HB level and systemic hypertension in LOS ≤ 7 days group showed significant differences compared to LOS > 7 days group, respectively.

**Table 1:** Baseline characteristics, operation-related and prognostic data of patients by LOS status (N = 311)

Characteristic	LOS ≤ 7 days (n = 226)	LOS > 7 days (n = 85)	*p-value*
Age (years) (mean ± SD)	60.14 ± 14.19	61.30 ± 14.42	0.930
Gender, n (%)			0.049
Male	102 (45.13)	49 (57.65)	
Female	124 (54.87)	36 (42.35)	
BMI (kg/m^2^) (mean ± SD)	31.37 ± 13.62	31.37 ± 13.85	0.458
SBP(mmHg) (mean ± SD)	125.92 ± 19.36	123.75 ± 23.91	0.435
DBP(mmHg) (mean ± SD)	70.35 ± 12.25	67.96 ± 16.43	0.829
Heart Rate(bpm) (mean ± SD)	74.93 ± 13.66	80.18 ± 16.55	0.006
Tobacco, n (%)	120 (53.10)	43 (50.59)	0.693
Yes			
ASA classification (%)			< 0.001
II	18 (7.96)	2 (2.35)	
III	169 (74.78)	50 (58.82)	
IV	39 (17.26)	33 (38.82)	
Category of surgery			
Intraabdominal, n (%)	49 (21.68)	28 (32.94)	0.040
Yes			
Intrathoracic, n (%)	13 (5.75)	14 (16.47)	0.003
Yes			
Suprainguinal Vascular, n (%)	13 (5.75)	2 (2.35)	0.212
Yes			
Intracranial (%)	3 (1.33)	1 (1.18)	0.916
Yes			
Systemic hypertension (%)	158 (69.91)	46 (54.12)	0.009
Yes			
Angina, n (%)	17 (7.52)	6 (7.06)	0.889
Yes			
Coronary artery disease, n (%)	58 (25.78)	29 (34.52)	0.128
Yes			
Congestive heart failure, n (%)	60 (26.55)	25 (29.41)	0.614
Yes			
Arrhythmia, n (%)	84 (37.17)	32 (37.65)	0.938
Yes			
Venous thromboembolism, n (%)	10 (4.42)	9 (10.59)	0.043
Yes			
Asthma, n (%)	30 (13.27)	9 (10.59)	0.524
Yes			
COPD, n (%)	32 (14.16)	16 (19.05)	0.290
Yes			
Obstructive sleep apnea, n (%)	48 (21.24)	20 (23.53)	0.663
Yes			
Diabetes, n (%)	67 (29.78)	21 (24.71)	0.377
Yes			
Renal failure (serum creatinine > 1.5 mg/dl), n (%)	55 (24.34)	23 (27.06)	0.622
Yes			
Anaemia, n (%)			
None	98 (43.36)	21 (24.71)	< 0.001
Mild	54 (23.89)	14 (16.47)	
Moderate/severe	74 (32.74)	50 (58.82)	
Serum creatinine (mg/dl), n (%)	1.00 (0.78 − 1.50)	1.13 (0.80 − 1.63)	0.211
Hemoglobin level(mg/dl) (mean ± SD)	12.04 ± 2.20	10.92 ± 2.22	< 0.001
White blood cell count(× 10^9^/l) (median [IQR])	7.52 (5.69–9.36)	7.23 (5.91–10.23)	0.505
Room Air SaO2, n (%)	189 (85.52)	72 (84.71)	0.857
Mortality, n (%)	1 (0.44)	6 (7.06)	< 0.001
Yes			
Procedure length (minutes) (Median [IQR])	87.00 (41.00–141.75)	135.00 (75.50–261.75)	< 0.001
*ECHO finding*			
Combined PASP and RVSP (mean ± SD)	42.56 ± 11.97	46.00 ± 12.37	0.006
*PHTN Severity Class*, n (%)			0.032
No PHTN	112 (51.61)	29 (34.94)	
Mild PHTN	89 (41.01)	47 (56.63)	
Moderate to severe PHTN	16 (7.37)	7 (8.43)	
EF value (mean ± SD)	59.40 ± 13.19	58.44 ± 14.46	0.601

*LOS* Length of Stay, *ASA* American Society of Anesthesiologists, *BMI* body mass index, *SBP* systolic blood pressure, *DBP* Diastolic Blood Pressure, *COPD* chronic obstructive pulmonary disease, *ECHO* echocardiography, *SD* Standard Deviation, *IQR* interquartile range, *PASP* pulmonary artery systolic pressure, *PHTN* pulmonary hypertension, *RAP* right atrial pressure, *RVSP* right ventricular systolic pressure, *EF* Left Ventricular Ejection Fraction

### Analysis of the associations between LOS and the clinical-pathological features of non-cardiac and non-obstetric patients

Preoperative anemia was present in 192 (61.74%) of 311 patients based on the WHO's gender-based criteria for anemia severity [14]. We observed that the ratio of moderate-to-severe anemia in LOS > 7 days group was higher compared to LOS ≤ 7 days group (58.82% vs. 32.74%, *p* < 0.05). The proportion of mortality patients was 0.44% in LOS ≤ 7 days group and 7.06% in LOS > 7 days group (*p* < 0.001). Whereas procedure length, heart rate, combined PASP and RVSP were lower in LOS ≤ 7 days group than that in LOS > 7 days group, respectively (*p* < 0.05). Moreover, characteristics including intra-abdominal, intrathoracic, and PHTN severity class exhibited statistical difference between the two groups.

Due to a lack of data in few covariates such as blood pressure and heart rate, we then used multiple interpolation technology to supplement it (as detailed in Additional file 2: Table S5 and Additional file 3: Table S6). Based on this, we performed a univariate analysis to determine the association between clinical characteristics and LOS. Results in Table 2 showed that age, gender, BMI, SBP, DBP, heart rate, white blood cell count, room air SaO_2_, combined PASP and RVSP, PHTN severity class, angina, coronary artery disease, congestive heart failure, arrhythmia, venous thromboembolism, asthma, COPD, obstructive sleep apnea, renal failure, diabetes, and serum creatinine were not associated with LOS. However, tobacco, systemic hypertension, gender, and HB were negatively associated with LOS. In contrast, heart rate, intraabdominal, intrathoracic, venous thromboembolism, ASA IV, combined PASP and RVSP, PHTN severity class, procedure length, and mortality were positively associated with LOS. In addition, univariate analysis showed that the procedure length was closely associated with LOS.

**Table 2:** Univariate analysis for LOS

Covariate	β (95% CI)	*p-*value
Age (years)	− 0.03 (− 0.05, 0.11)	0.460
Gender		
Male	Reference	
Female	− 2.57 (− 4.71, − 0.43)	0.019
BMI (kg/m^2^)	− 0.03(− 0.11, 0.05)	0.443
SBP (mmHg)	− 0.02 (− 0.08, 0.03)	0.433
DBP (mmHg)	− 0.05 (− 0.13, 0.04)	0.267
Heart Rate	0.11 (− 0.03, 0.18)	0.007
Tobacco	Reference	
	− 1.57 (− 3.72, − 0.59)	0.155
ASA classification (%)		
II	Reference	
III	2.61 (− 1.71, 6.93)	0.238
IV	7.80 (3.12, 12.48)	0.001
Category of surgery		
Intraabdominal	Reference	
	19.81 (12.87,26.75)	< 0.0001
Intrathoracic	Reference	
	5.52 (1.74, 9.31)	0.005
Suprainguinal Vascular	Reference	
	− 0.23 (− 5.27, 4.81)	0.929
Intracranial	Reference	
	− 2.96 (− 12.55, 6.62)	0.545
Systemic hypertension	Reference	
	− 3.62 (− 5.86, − 1.38)	0.002
Angina	Reference	
	0.44 (− 3.69, 4.57)	0.834
Coronary artery disease	Reference	
	1.60 (− 0.81,4.01)	0.195
Congestive heart failure	Reference	
	1.41 (− 1.01, 3.83)	0.254
Arrhythmia	Reference	
	0.58 (− 1.65, 2.82)	0.610
Venous thromboembolism	Reference	
	4.92 (0.45, 9.40)	0.032
Asthma	Reference	
	− 3.03 (− 6.28, 0.21)	0.068
COPD	Reference	
	0.53 (− 2.47, 3.52)	0.730
Obstructive sleep apnea	Reference	
	− 0.09 (− 2.70, 2.53)	0.947
Diabetes	Reference	
	− 1.01 (− 3.41, 1.39)	0.410
Renal failure (serum creatinine > 1.5 mg/dl)	Reference	
	2.41 (0.07, 4.89)	0.058
HB(g/dl)	− 1.18 (− 1.64, − 0.71)	< 0.0001
White blood cell count(× 10^9^/l)	− 0.01 (− 0.09, 0.08)	0.861
Serum creatinine	0.53 (− 0.14, 1.20)	0.120
Room Air SaO2	Reference	
	− 1.89 (− 4.97, 1.19)	0.231
Mortality	Reference	
	19.81 (12.87, 26.75)	< 0.001
Procedure length	0.02 (0.02, 0.03)	< 0.0001
*ECHO Finding*		
Combined PASP and RVSP	0.13(− 0.04, 0.22)	0.007
PHTN Severity Class		
No PHTN	Reference	
Mild PHTN	2.39 (0.09, 4.68)	0.042
Moderate to severe PHTN	4.41 (0.13, 8.70)	0.045
EF Value	0.01 (− 0.09, 0.07)	0.786

*CI* Confidence Interval, *LOS* Length Of Stay, *ASA* American Society of Anesthesiologists, *BMI* body mass index, *SBP* systolic blood pressure, *DBP* Diastolic Blood Pressure, *COPD* chronic obstructive pulmonary disease, *ECHO* echocardiography, *IQR* interquartile range, *PASP* pulmonary artery systolic pressure, *PHTN* pulmonary hypertension, *RAP* right atrial pressure, *RVSP* right ventricular systolic pressure, *EF* Left Ventricular Ejection Fraction

### Association between preoperative HB level and LOS in non-cardiac and non-obstetric patients

To better clarify the association between preoperative HB level and LOS in non-cardiac, non-obstetric surgery patients, we further conducted multiple regression analysis. Results of three models (non-adjusted model, Model I, and Model II) in Table 3 and 4 indicated that there was a negative association between preoperative HB level and LOS in non-cardiac, non-obstetric surgery patients, respectively (*p* < 0.0001).

**Table 3:** Non-adjusted and adjusted linear regression models

Outcome	Non-adjusted model		Model I		Model II	
	β (95% CI)	*p*-value	β (95% CI)	*p*-value	β (95% CI)	*p*-value
Hemoglobin level	− 1.18 (− 1.64, − 0.71)	< 0.0001	− 0.99 (− 1.45, − 0.54)	< 0.0001	− 0.86 (− 1.31, − 0.41)	< 0.0002

Non-adjusted model adjust for: None

Model I adjust for: mortality

Model II adjust for: Gender, ASA classification, procedure length, Combined PASP and RVSP, mortality, Intrathoracic, systemic hypertension, Venous thromboembolism, Asthma, Renal failure (serum creatinine > 1.5 mg/dl), and Heart Rate

*CI* Confidence Interval, *LOS* length of stay, *HB* hemoglobin, *ASA* American Society of Anesthesiologists, *PASP* pulmonary artery systolic pressure, *RVSP* right ventricular systolic pressure

**Table 4:** Non-adjusted and adjusted logistic regression models

Outcome	Non-adjusted model		Model I		Model II	
	OR (95% CI)	*p*-value	OR (95% CI)	*p*-value	OR (95% CI)	*p*-value
Hemoglobin level	0.79 (0.70, 0.89)	< 0.0001	0.81(0.72, 0.91)	< 0.0007	0.76 (0.65, 0.90)	< 0.0010

Non-adjusted model adjust for: None

Model I adjust for: mortality

Model II adjust for: Gender, ASA classification, procedure length, Combined PASP and RVSP, mortality, Intrathoracic, systemic hypertension, Venous thromboembolism, Asthma, Renal failure (serum creatinine > 1.5 mg/dl), and Heart Rate

*CI* Confidence Interval, *OR* Odds Ratio, *LOS* Length of Stay, *HB* hemoglobin, *ASA* American Society of Anesthesiologists, *PASP* pulmonary artery systolic pressure, *RVSP* right ventricular systolic pressure

For missing data on some covariates, we used multiple interpolation techniques to supplement and maximize statistical effects, and conducted a sensitivity analysis on the data. The results showed that the complete data analysis results after imputation were consistent with the multiple regression results excluding missing data (Additional file 2: Tables S5 and Additional file 3: Table S6).

**Fig. 2:**
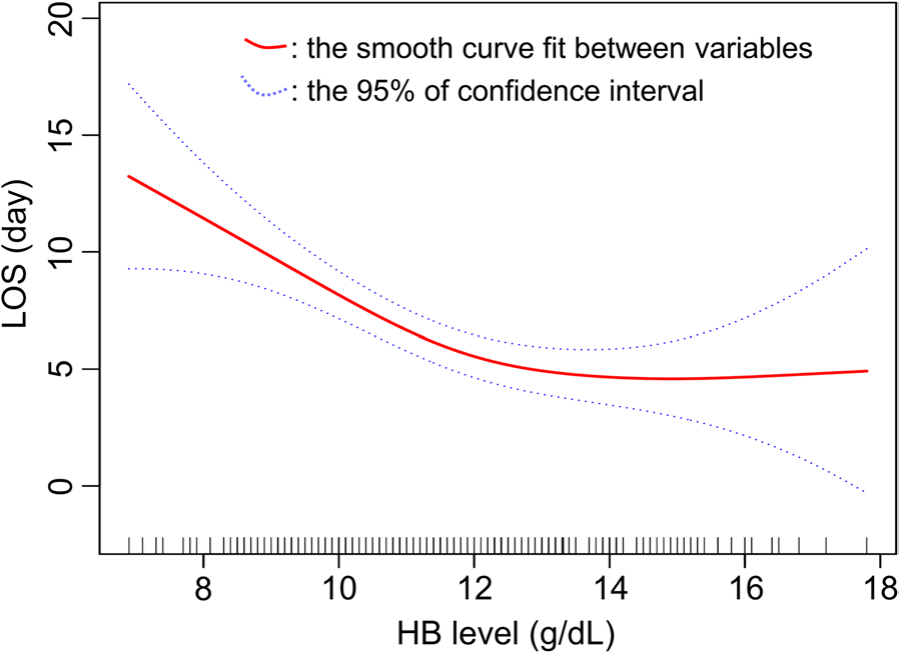
Association between HB and LOS. A threshold, nonlinear association between HB and LOS was found in a generalized additive model (GAM). Solid red line represents the smooth curve fit between variables. Blue bands represent the 95% of confidence interval from the fit. All adjusted for Heart Rate, tobacco, Renal failure (serum creatinine > 1.5 mg/dl), ASA classification, procedure length, mortality, Intrathoracic, systemic hypertension, and DBP

Additionally, we found a nonlinear relation between preoperative HB level and LOS by cubic spline smoothing technique, and such a relationship still existed after adjusting for the confounding factors (Fig. 2). The inflection point of HB level was calculated to be 11.9 g/dL (Table 5). When preoperative HB level < 11.9 g/dL, LOS decreased by 2 days for per 1 g/dL increase in HB level. However, when preoperative HB level > 11.9 g/dL, the estimated dose–response curve was consistent with a horizontal line, suggesting a non-significant relation between HB level and LOS.

**Table 5:** Threshold Effect Analysis of HB and LOS using Piece-wise Linear Regression

Inflection point of HB	β (95% CI)	*p*-value
< 11.9	− 2.00 (− 2.92, − 1.08)	< 0.0001
> 11.9	0.21 (− 0.67, 1.08)	0.641

Effect: LOS, Cause: HB

Adjusted: Gender, ASA classification, procedure length, Combined PASP and RVSP, mortality, intrathoracic, systemic hypertension, Venous thromboembolism, Asthma, Renal failure (serum creatinine > 1.5 mg/dl), and Heart Rate

*CI* Confidence Interval, *LOS* Length of Stay, *HB* hemoglobin, *ASA* American Society of Anesthesiologists, *PASP* pulmonary artery systolic pressure, *RVSP* right ventricular systolic pressure

## Discussion

This retrospective cohort study investigated the effect of preoperative HB level on LOS in non-cardiac and non-obstetric patients. By adjusting for the potential risk factors related to LOS, we showed that patients with prolonged hospitalization had a low preoperative HB level and a high prevalence of moderate-to-severe anemia, which were consistent with previous results [7, 8, 17]. These results indicate that more attention should be paid to this group of patients, so as to detect anemia-related symptoms in time and provide iron supplements, vitamin B12, and blood transfusion treatment when necessary.

In addition, we found that the incidence of preoperative anemia was 61.7% in non-cardiac and non-obstetric patients, and the incidence of mild and moderate-to-severe anemia was 21.9% and 39.9%, respectively. These results were in conflict with previous studies which indicated that the incidence of anemia in patients without cardiac surgery was 28.7–30.44% [4, 5]. Such a discrepancy could be explained by different patient characteristics and the definition criteria for anemia in different studies. Indeed, some researchers employed hemoglobin levels to define anemia, while others used hematocrit values.

Clinically, a lack of hematopoietic raw materials, including iron or folic acid, vitamin B12, and chronic diseases such as chronic infection, tumors, chronic renal insufficiency, etc., may lead to preoperative anemia [18]. In addition, some patients may experience unexplained anemia owing to multiple complex mechanisms. If such a symptom is not treated promptly and efficiently, the procedure and prognosis may be compromised. For example, Rasouli M.R. et al. found that patients undergoing total joint arthroplasties presented the highest (4.23%) infection rate with preoperative HB 10 g/dL, whereas the lowest (0.84%) infection rate with preoperative HB of 12 ~ 13 g/dL [19]. Moreover, preoperative anemia might increase postoperative mortality, the occurrence of complications, and negatively impact patients' postoperative activities and functional recovery [9, 20]. All of the above factors will lead to prolonged hospital stays and increased costs, which in turn highlights the importance of shortening hospital stays, as it can not only reduce medical insurance costs and save medical and health resources, but also increase bed turnover and reduce waiting times for elective surgeries.

Previous research has shown a linear relationship between preoperative anemia and LOS, with one study suggesting that LOS would decrease by 0.2 days for every 1 g/dL increase in preoperative HB [17]. In this study, we discovered a nonlinear association between preoperative HB level and LOS as well as a threshold effect based on LOS after controlling for relevant confounders. Specifically, when preoperative HB level was less than 11.9 g/dL, LOS decreased by 2 days for each 1 g/dL increase in HB. However, LOS did not alter substantially with the rise of preoperative HB level when it was larger than 11.9 g/dL. Even though the results of this study on the relation between preoperative HB levels and LOS are inconsistent with previous studies. However, paying attention to the patient's anemia symptoms before and after surgery and providing timely treatment will be useful to reduce the patient's hospitalization time to a certain extent. At the same time, for patients undergoing non-cardiac and non-obstetric surgery, when the preoperative HB level is greater than 11.9 g/dL, adjusting the lipid level or blood glucose level may be also good for reducing the length of hospital stay, which requires further in-depth research.

It is necessary to point out the limitations of this retrospective study. First, some data were excluded due to the absence of certain preoperative HB level, resulting in a reduction in sample size and selection bias. Second, the clinical data came from a single center, leading to a lack of data on some covariates, such as treatment technology and ethnic differences. Consequently, a larger, multicenter investigation of individuals undergoing non-cardiac surgery and non-obstetric surgery may be necessary.

## Conclusion

This study showed that preoperative anemia is prevalent among patients undergoing non-cardiac and non-obstetric surgery. The LOS was closely connected with the preoperative HB level, with a threshold and saturation effect. Therefore, careful monitoring and treatment of patients with preoperative anemia and adjustment of preoperative HB level to 11.9 g/dL or higher are important, which may help to shorten the LOS and reduce medical costs.

### Supplementary Information

**Additional file 1: **The specific screening process of this study.

**Additional file 2: **The relationship between HB and LOS analyzed based on the data containing missing data and multiple interpolation.

**Additional file 3: **The relationship between HB and LOS > 7days analyzed based on the data containing missing data and multiple interpolation.

## Data Availability

Data can be down-loaded from “DATADRYAD” database. Dryad data package: Shah, Aalap C. et al. (2019), Data from: Self-reported functional status predicts post-operative outcomes in non-cardiac surgery patients with pulmonary hypertension, Dryad, Dataset, https://doi.org/10.5061/dryad.9236ng5.
