# Supplementary material for: Association between preoperative hemoglobin with length of hospital stay among non-cardiac and non-obstetric surgery patients: a secondary analysis of a retrospective cohort study
Source: J Cardiothorac Surg. 2024 Feb 16;19:97. doi: 10.1186/s13019-024-02566-5 (PMC10870523; doi:10.1186/s13019-024-02566-5)
Supplement: Supplementary file 1 — Additional file 1: The specific screening process of this study. [file 13019_2024_2566_MOESM1_ESM.docx]

Supplementary table 1. Covariate examination and screening: VIF collinearity screen.

|  | Step 1 |
| --- | --- |
| HB (g/dl) | 1.3 |
| Age (years) | 1.5 |
| Gender | 1.3 |
| BMI (kg/m^2^) | 1.5 |
| ASA classification (%) | 1.4 |
| tobacco | 1.3 |
| Procedure length | 1.4 |
| Combined PASP and RVSP | 1.2 |
| Mortality | 1.2 |
| Intraabdominal | 1.5 |
| Intrathoracic | 1.3 |
| Suprainguinal Vascular | 1.2 |
| Intracranial | 1.1 |
| Systemic hypertension | 1.4 |
| Coronary artery disease | 1.5 |
| Congestive heart failure | 1.4 |
| Venous thromboembolism | 1.1 |
| Arrhythmia | 1.4 |
| Asthma | 1.2 |
| COPD | 1.3 |
| Obstructive sleep apnea | 1.5 |
| Diabetes | 1.3 |
| Renal failure (serum creatinine > 1.5 mg/dl) | 1.7 |
| Angina | 1.3 |
| SBP (mmHg) | 2.3 |
| DBP (mmHg) | 2.2 |
| Heart Rate | 1.4 |
| Room Air SaO2 | 1.2 |
| Serum creatinine | 1.8 |
| white blood cell count (×10^9^/l) | 1.2 |

Abbreviations: VIF: Variance inflation factor, HB: hemoglobin, BMI: body mass index, ASA: American Society of Anesthesiologists, PASP: pulmonary artery systolic pressure, RVSP: right ventricular systolic pressure, COPD: chronic obstructive pulmonary disease, SBP: preoperative systolic, DBP: diastolic blood pressure.

Supplementary table 2. The relationship between covariates and LOS

| Covariates | N | term | β | Se. | 95%CI Low | 95%CI Upp | P value |
| --- | --- | --- | --- | --- | --- | --- | --- |
| Age (years) | 308 | Age | 0.0289 | 0.0391 | -0.0478 | 0.1056 | 0.4602 |
| Gender | 311 | Factor (Gender)2 | -2.5717 | 1.0933 | -4.7146 | -0.4287 | 0.0193 |
| BMI (kg/m^2^) | 308 | BMI | -0.0313 | 0.0407 | -0.1111 | 0.0485 | 0.4428 |
| ASA classification | 311 | Factor (ASA classification)3 | 2.6091 | 2.2059 | -1.7144 | 6.9327 | 0.2378 |
|  |  | Factor (ASA classification)4 | 7.7975 | 2.3869 | 3.1191 | 12.4758 | 0.0012 |
| Tobacco | 311 | Factor (TOBACCO)1 | -1.5678 | 1.1003 | -3.7244 | 0.5888 | 0.1552 |
| Proc Length | 308 | Proc Length | 0.0247 | 0.0045 | 0.0160 | 0.0335 | <0.0001 |
| Combined PASP and RVSP | 300 | Combined PASP and RVSP | 0.1257 | 0.0461 | 0.0354 | 0.2161 | 0.0068 |
| Mortality | 311 | Factor (Mortality)1 | 19.8126 | 3.5418 | 12.8707 | 26.7545 | <0.0001 |
| Intraabdominal | 311 | Factor (Intraabdominal)1 | 1.4280 | 1.2747 | -1.0705 | 3.9265 | 0.2635 |
| Intrathoracic | 311 | Factor (Intrathoracic)1 | 5.5238 | 1.9326 | 1.7359 | 9.3118 | 0.0045 |
| Suprainguinal Vascular | 311 | Factor (Suprainguinal Vascular)1 | -0.2307 | 2.5731 | -5.2740 | 4.8126 | 0.9286 |
| Intracranial | 311 | Factor (Intracranial)1 | -2.9614 | 4.8899 | -12.5455 | 6.6228 | 0.5452 |
| Systemic Hypertension | 311 | Factor (Systemic Hypertension)1 | -3.6218 | 1.1421 | -5.8603 | -1.3834 | 0.0017 |
| Coronary Artery Disease | 309 | Factor (coronary artery disease)1 | 1.5986 | 1.2293 | -0.8109 | 4.0081 | 0.1945 |
| Congestive heart failure | 311 | Factor (Congestive heart failure)1 | 1.4118 | 1.2345 | -1.0078 | 3.8313 | 0.2537 |
| Venous thromboembolism | 311 | Factor (Venous thromboembolism)1 | 4.9233 | 2.2848 | 0.4451 | 9.4015 | 0.0319 |
| Arrhythmia | 311 | Factor (Arrhythmia )1 | 0.5823 | 1.1395 | -1.6512 | 2.8158 | 0.6097 |
| Asthma | 311 | Factor (Asthma)1 | -3.0335 | 1.6557 | -6.2788 | 0.2118 | 0.0679 |
| COPD | 310 | Factor (COPD)1 | 0.5268 | 1.5275 | -2.4671 | 3.5207 | 0.7304 |
| Obstructive sleep apnea | 311 | Factor (Obstructive sleep apnea)1 | -0.0880 | 1.3338 | -2.7023 | 2.5263 | 0.9474 |
| Diabetes | 310 | Factor (Diabetes)1 | -1.0106 | 1.2251 | -3.4119 | 1.3906 | 0.4100 |
| Renal failure (serum creatinine > 1.5 mg/dl) | 311 | Factor (Renal failure )1 | 2.4089 | 1.2644 | -0.0694 | 4.8872 | 0.0577 |
| Angina | 311 | Factor (Angina)1 | 0.4413 | 2.1065 | -3.6875 | 4.5700 | 0.8342 |
| SBP (mmHg) | 283 | SBP | -0.0219 | 0.0279 | -0.0765 | 0.0327 | 0.4328 |
| DBP (mmHg) | 283 | DBP | -0.0475 | 0.0427 | -0.1311 | 0.0362 | 0.2672 |
| Heart Rate | 290 | Heart Rate | 0.1073 | 0.0394 | 0.0301 | 0.1844 | 0.0068 |
| Room Air SaO2 | 306 | Factor (Room Air SaO2)1 | -1.8900 | 1.5739 | -4.9748 | 1.1949 | 0.2308 |
| Serum creatinine | 295 | Serum creatinine | 0.5340 | 0.3422 | -0.1368 | 1.2048 | 0.1198 |
| white blood cell count (×10^9^/L) | 307 | white blood cell count | -0.0075 | 0.0428 | -0.0913 | 0.0763 | 0.8609 |

Abbreviations: BMI: body mass index, ASA: American Society of Anesthesiologists, PASP: pulmonary artery systolic pressure, RVSP: right ventricular systolic pressure, COPD: chronic obstructive pulmonary disease, SBP: preoperative systolic, DBP: diastolic blood pressure.

Supplementary table 3. Introduction of covariates into the basic model and elimination of covariates from the complete model to determine changes in the regression coefficient of HB (g/dl)

|  |  | Basic model | Complete model |  |
| --- | --- | --- | --- | --- |
| Covariate | +/- term | HB(g/dl) | HB(g/dl) | Selected |
|  | Initial regression coefficient | -1.2741 | -1.0736 |  |
| Age (years) | Age | -1.2629 | -1.0847 |  |
| Gender | Factor (Gender) | -1.2646 | -1.0809 |  |
| BMI (kg/m^2^) | BMI | -1.2748 | -1.0688 |  |
| ASA classification | Factor (ASA classification) | -1.2167 | -1.0720 |  |
| Tobacco | Factor (Tobacco) | -1.2660 | -1.0822 |  |
| Proc Length | Proc Length | -1.2419 | -1.0585 |  |
| Combined PASP and RVSP | Combined PASP and RVSP | -1.2490 | -1.0898 |  |
| Mortality | Factor (Mortality) | -0.9931 * | -1.2825 * | Yes |
| Intraabdominal | Factor (Intraabdominal) | -1.2783 | -1.0733 |  |
| Intrathoracic | Factor (Intrathoracic) | -1.2050 | -1.1065 |  |
| Suprainguinal Vascular | Factor (Suprainguinal Vascular) | -1.2793 | -1.0692 |  |
| Intracranial | Factor (Intracranial) | -1.2721 | -1.0751 |  |
| Systemic Hypertension | Factor (Systemic Hypertension) | -1.3193 | -0.9952 |  |
| Coronary Artery Disease | Factor (coronary artery disease) | -1.2649 | -1.0720 |  |
| Congestive heart failure | Factor (Congestive heart failure) | -1.2898 | -1.0651 |  |
| Venous thromboembolism | Factor (Venous thromboembolism) | -1.2658 | -1.0516 |  |
| Arrhythmia | Factor (Arrhythmia) | -1.3033 | -1.0578 |  |
| Asthma | Factor (Asthma) | -1.2596 | -1.0793 |  |
| COPD | Factor (COPD) | -1.2763 | -1.0739 |  |
| Obstructive sleep apnea | Factor (Obstructive sleep apnea) | -1.2953 | -1.0426 |  |
| Diabetes | Factor (Diabetes) | -1.3159 | -1.0568 |  |
| Renal failure (serum creatinine > 1.5 mg/dl) | Factor (Renal failure) | -1.2047 | -1.1235 |  |
| Angina | Factor (Angina) | -1.2718 | -1.0751 |  |
| SBP (mmHg) | SBP | -1.2719 | -1.0716 |  |
| DBP (mmHg) | DBP | -1.2723 | -1.0632 |  |
| Heart Rate | Heart Rate | -1.2271 | -1.1236 |  |
| Room Air SaO2 | Factor (Room Air SaO2) | -1.3396 | -1.0061 |  |
| Creatinine | Creatinine | -1.2571 | -1.0663 |  |
| white blood cell count (×10^9^/L) | white blood cell count | -1.2980 | -1.0391 |  |

* The change in the initial regression coefficient was more than 10%

Abbreviations: BMI: body mass index, ASA: American Society of Anesthesiologists, PASP: pulmonary artery systolic pressure, RVSP: right ventricular systolic pressure, COPD: chronic obstructive pulmonary disease, SBP: preoperative systolic, DBP: diastolic blood pressure.

Supplementary table 4. Filtered covariates

| Y | X | Selected covariates (Criterion 1) | Selected covariates (Criterion 2) |
| --- | --- | --- | --- |
| LOS | HB | mortality | Gender, ASA classification, procedure length, Combined PASP and RVSP, mortality, Intrathoracic, systemic hypertension, Venous thromboembolism, Asthma, Renal failure (serum creatinine > 1.5 mg/dl), Heart Rate |

**Annotation:**

1. Standard 1: The effect of introducing covariables in the basic model or removing covariables in the complete model on the regression coefficient of X >10%

2. Standard 2: Standard 1 or regression coefficient P value of covariate to Y <0.1
