# Supplementary material for: Association between preoperative hemoglobin with length of hospital stay among non-cardiac and non-obstetric surgery patients: a secondary analysis of a retrospective cohort study
Source: J Cardiothorac Surg. 2024 Feb 16;19:97. doi: 10.1186/s13019-024-02566-5 (PMC10870523; doi:10.1186/s13019-024-02566-5)
Supplement: Supplementary file 2 — Additional file 2: The relationship between HB and LOS analyzed based on the data containing missing data and multiple interpolation. [file 13019_2024_2566_MOESM2_ESM.docx]

Supplementary Table 5. Based on the data containing missing data and multiple interpolation, relation between HB and LOS

| Outcome |  | With missing data | |  | Multiple imputation | |
| --- | --- | --- | --- | --- | --- | --- |
|  |  | β (95%CI) | P-value |  | β (95%CI) | P-value |
| Hemoglobin level |  | -0.86 (-1.31, -0.41) | <0.0002 |  | -0.872（-1.283,-0.461） | <0.0001 |

Abbreviations: CI: Confidence Interval.

The two groups of data were adjusted variables including Gender, ASA classification, procedure length, Combined PASP and RVSP, mortality, Intrathoracic, systemic hypertension, Venous thromboembolism, Asthma, Renal failure (serum creatinine > 1.5 mg/dl), and Heart Rate.
